# A novel c-Met inhibitor, MK8033, synergizes with carboplatin plus paclitaxel to inhibit ovarian cancer cell growth

**DOI:** 10.3892/or.2013.2329

**Published:** 2013-03-05

**Authors:** DOUGLAS C. MARCHION, ELONA BICAKU, YIN XIONG, NADIM BOU ZGHEIB, ENTIDHAR AL SAWAH, XIAOMANG BA STICKLES, PATRICIA L. JUDSON, ALEX S. LOPEZ, CHRISTOPHER L. CUBITT, JESUS GONZALEZ-BOSQUET, ROBERT M. WENHAM, SACHIN M. APTE, ANDERS BERGLUND, JOHNATHAN M. LANCASTER

**Affiliations:** 1 Department of Women's Oncology, H. Lee Moffitt Cancer Center and Research Institute, Tampa, FL 33612, USA; 2 Experimental Therapeutics Program, H. Lee Moffitt Cancer Center and Research Institute, Tampa, FL 33612, USA; 3 Department of Anatomic Pathology, H. Lee Moffitt Cancer Center and Research Institute, Tampa, FL 33612, USA; 4 Department of Oncologic Sciences, H. Lee Moffitt Cancer Center and Research Institute, Tampa, FL 33612, USA; 5 Translational Research Core, H. Lee Moffitt Cancer Center and Research Institute, Tampa, FL 33612, USA; 6 Cancer Informatics Core, H. Lee Moffitt Cancer Center and Research Institute, Tampa, FL 33612, USA

**Keywords:** c-Met expression, combination index, ovarian cancer, carboplatin, immunohistochemistry

## Abstract

Elevated serum levels of hepatocyte growth factor (HGF) and high tumor expression of c-Met are both indicators of poor overall survival from ovarian cancer (OVCA). In the present study, we evaluated the role of the HGF signaling pathway in OVCA cell line chemoresistance and OVCA patient overall survival as well as the influence of HGF/c-Met signaling inhibition on the sensitivity of OVCA cells to combinational carboplatin plus paclitaxel therapy. The prevalence of the HGF receptor, c-Met, was determined by immunohistochemistry in primary OVCA samples (n=79) and OVCA cell lines (n=41). The influence of the c-Met-specific inhibitor MK8033 on OVCA cell sensitivity to combinations of carboplatin plus paclitaxel was examined in a subset of OVCA cells (n=8) by CellTiter-Blue cell viability assays. Correlation tests were used to identify genes associated with response to MK8033 and carboplatin plus paclitaxel. Identified genes were evaluated for influence on overall survival from OVCA using principal component analysis (PCA) modeling in an independent clinical OVCA dataset (n=218). Immunohistochemistry analysis indicated that 83% of OVCA cells and 92% of primary OVCA expressed the HGF receptor, c-Met. MK8033 exhibited significant anti-proliferative effects against a panel of human OVCA cell lines. Combination index values determined by the Chou-Talalay isobologram equation indicated synergistic activity in combinations of MK8033 and carboplatin plus paclitaxel. Pearson's correlation identified a 47-gene signature to be associated with MK8033-carboplatin plus paclitaxel response. PCA modeling indicated an association of this 47-gene response signature with overall survival from OVCA (P=0.013). These data indicate that HGF/c-Met pathway signaling may influence OVCA chemosensitivity and overall patient survival. Furthermore, HGF/c-Met inhibition by MK8033 represents a promising new therapeutic avenue to increase OVCA sensitivity to carboplatin plus paclitaxel.

## Introduction

Ovarian cancer (OVCA) is the fifth leading cause of cancer-related mortality among women in the United States and Western Europe, with the highest mortality rate of all gynecologic malignancies. Approximately 75% of patients with OVCA are diagnosed at an advanced stage (III/IV) with disseminated intraperitoneal metastases (1). Although the majority of patients initially experience a complete clinical response to primary surgery followed by platinum/taxane-based chemotherapy, most patients eventually develop platinum/taxane-resistant persistent or recurrent disease. These patients have a poor prognosis. Despite cytoreductive surgery and aggressive chemotherapy, the majority of patients with OVCA succumb to the disease within 5 years (2). These dismal statistics highlight the need for research into the cellular basis of platinum response, as well as the design of targeted therapeutic strategies.

The hepatocyte growth factor (HGF) signaling pathway may be a viable target for the development of directed therapeutic regimens for the treatment of OVCA. HGF is the only known ligand of the HGF receptor (c-Met), which is expressed in approximately 70% of OVCAs (3,4). Overexpression of HGF and/or c-Met has been associated with poor clinical outcome in OVCA (5–7), whereas c-Met overexpression has further been associated with a chemo-resistant subset of ovarian clear-cell adenocarcinomas (8,9). The targeted inhibition of c-Met has been shown to reduce c-Met expression, block OVCA cell proliferation, and reduce tumor burden in pre-clinical mouse models (10–12).

In this study, we analyzed the expression of the HGF receptor, c-Met, in primary OVCA and OVCA cell lines, and tested the activity of the HGF/c-Met signaling inhibitor, MK8033, alone and in combination with standard of care therapy (carboplatin plus paclitaxel) in a panel of OVCA cells. We then identified the genes associated with sensitivity to MK8033 plus carboplatin-paclitaxel and used the principal component analysis (PCA) to define a gene expression signature of OVCA cell response. Finally, this PCA response signature was tested in an independent survival dataset of primary OVCA for which response to chemotherapy and overall survival were known.

## Materials and methods

### Cell culture

OVCA cell lines were obtained from the American Type Culture Collection (Manassas, VA, USA) (CAOV3, OV90, OVCAR3 and SKOV3); from the European Collection of Cell Cultures (Salisbury, England) (A2780CP and A2780S); from Kyoto University (Japan) (CHI, CHIcisR, M41, M41CSR, Tyknu and TyknuCisR); or were kind gifts from Dr Patricia Kruk, Department of Pathology, College of Medicine, University of South Florida, Tampa, FL, and Professor Susan Murphy, Department of OBGYN/Division of Gynecologic Oncology, Duke University, Durham, NC, USA (A2008, C13, CAOV2, HeyA8, IGR-OV1, IMCC3, IMCC5, MCAS, OV2008, OVCA420, OVCA429, OVCA432, OVCA433, FUOV1, PEO1, PEO4, SK-OV-6, T8, TOV-112D, TOV-21-G, Dov13, BG1, Ovary1847, OVCAR10, OVCAR8, OVCAR5, OVCAR4, OVCAR2 and SK-OV-4). Cell lines were maintained in RPMI-1640 (Invitrogen, Carlsbad, CA, USA) supplemented with 10% fetal bovine serum (Fisher Scientific, Pittsburg, PA, USA), 1% sodium pyruvate, 1% penicillin/streptomycin (Cellgro, Manassas, VA, USA), and 1% nonessential amino acids (HyClone, Hudson, NH, USA). Mycoplasma testing was performed every 6 months in accordance with the manufacturer's protocol (Lonza, Rockland, ME, USA).

### Staining and scoring of c-Met expression in OVCA

Slides were stained using a Ventana Discovery XT automated system as per the manufacturer's protocol with proprietary reagents (Ventana Medical Systems, Tucson, AZ, USA). Briefly, slides were deparaffinized on the automated system with EZ Prep solution (Ventana). The heat-induced antigen retrieval method was used in Cell Conditioning 1 (Ventana). The mouse monoclonal antibody that reacts to c-Met (#18-7366; Invitrogen) was used at a 1:2,000 concentration in PSS antibody diluent (Ventana) and incubated for 60 min. The Ventana OmniMap anti-mouse secondary antibody was used for 16 min. The detection system used was the Ventana ChromoMap kit, and slides were counterstained with hematoxylin. Slides were then dehydrated and coverslipped as per normal laboratory protocol. The c-Met expression score was defined as the product of intensity and cellularity, where an intensity of 1 was weak; 2, moderate; 3, strong, and a cellularity of 1 ≤33%, 2 =34–65% and 3 ≥66%.

### RNA extraction and microarray expression analysis

RNA extraction and microarray expression analyses were performed as previously described (13). Briefly, RNA was extracted using the RNeasy kit following the manufacturer's recommendations (Qiagen, Valencia, CA, USA). Quality of the RNA was measured using an Agilent 2100 Bioanalyzer. The targets for Affymetrix DNA microarray analysis were prepared according to the manufacturer's instructions. For OVCA cell lines (n=41), targets were hybridized to customized Human Affymetrix HuRSTA gene chips (HuRSTA-2a520709) [GEO accession number GSE34615 (14)].

### Statistical analyses

Expression data were subjected to background correction and normalization using the Robust Multichip Average algorithm in the Affymetrix Expression Console (http://www.affymetrix.com/products/software/specific/expression_console_software.affx). Pearson's correlation test was performed on individual gene expression and IC_50_ values. Probe sets with P<0.001 were considered to have significant correlations with IC_50_ values.

### Cell viability assays

For cells cultured in the presence of cisplatin, resistance was quantified using MTT proliferation assay, in accordance with manufacturer's instructions (Promega, Madison, WI, USA). Cells (5,000/well) were seeded in 96-well plates, allowed to adhere, and incubated with increasing doses of cisplatin for 48 h at 37°C. Following drug incubation, plates were read at 570 nm using a SpectraMax 190 microplate reader (Molecular Devices, USA). For drug combination experiments, CellTiter-Blue cell viability assays were performed using 384-well plates and an automated pipetting station. Briefly, for these assays, 1.2×10^3^ cells (24-μl volume) were plated in each well and allowed to adhere overnight at 37°C and 5% CO_2_. Drug dilutions were then added using the same pipetting station, and the plate was incubated for 72 h. Following drug incubation, fluorescence was measured using a Synergy 4 microplate reader (Bio-Tek Instruments, Inc.). The fluorescence data were transferred to a spreadsheet program to calculate the percent viability relative to untreated cells. Experiments were repeated a minimum of three times.

### Synergism analyses

Drug combination experiments were analyzed for synergistic, additive, or antagonistic effects using the combination index method developed by Chou and Talalay. For the application of this method, the drug concentration dilutions of the drugs were used at fixed dose ratios based on the IC_50_ values of each drug obtained from preliminary experiments (e.g., 50:1, 2:5, 1:250). Briefly, the dose-effect curve for each drug alone is determined based on experimental observations using the median-effect principle and is compared to the effect achieved with a combination of the two drugs to derive a combination index value. This method involves plotting dose-effect curves, for each agent and their combination, using the following median-effect equation: fa/fu = (D/Dm)m, where D is the dose of the drug, Dm is the dose required for a 50% effect (equivalent to IC_50_), fa and fu are the affected and unaffected fractions, respectively (fa=1-fu), and m is the exponent signifying the sigmoidicity of the dose-effect curve. The computer software program XLfit was used to calculate the values of Dm and m. The combination index values used for the analysis of the drug combinations were determined by the isobologram equation for mutually nonexclusive drugs that have different modes of action: combination index = (D)1/(Dx)1 + (D)2/(Dx)2 + (D)1(D)2/(Dx)1(Dx)2, where (Dx)1 and (Dx)2 in the denominators are the doses (or concentrations) for D1 (drug 1) and D2 (drug 2) alone that gives x% inhibition, whereas (D)1 and (D)2 in the numerators are the doses of drugs 1 and 2 in combination that also inhibited x% (i.e., isoeffective). Combination indices <1, equal to 1, and >1 indicate synergism, additive effects and antagonism, respectively.

### Building signatures

The PCA methodology was used to derive a gene expression signature with a corresponding score. First, the data were reduced into a small set of uncorrelated principal components. This set of principal components was generated based on its ability to account for variation. The first principal component (1st PCA) is used to represent the overall variability in the data. The gene expression score is equal to ∑*w_i_x_i_*, a weighted average expression among the differentially expressed genes, where *x_i_* represents gene *i* expression level, *w_i_* is the corresponding weight (loading coefficient) with ∑*w^2^_i_* = 1, and the *w_i_* values maximize the variance of ∑*w_i_x_i_*. Directional signs of PCA scores are recognized to be arbitrary and can vary between software and the algorithm used to calculate the PCA model (15); however, this does not affect the interpretation of the PCA model and can be easily solved by multiplying both scores and loadings by −1, a 180° rotation. Details of this methodology have been reported by our group previously (13).

### Associations with overall survival from OVCA

PCA models were then explored for associations with overall survival from OVCA (using median PC1 score as the threshold to define high vs. low pathway score) using a dataset for which both gene expression and overall survival data were available, the publicly available Australian (Aus) Dataset (Affymetrix U133Plus GeneChips, n=218).

## Results

### c-Met is significantly expressed in OVCA

Since overexpression of HGF has been associated with poor clinical outcome in OVCA (5–7), we evaluated the expression of c-Met, the only known HGF receptor, in OVCA cells and primary OVCA tumor samples. Pathological scoring of c-Met immunostains (cellularity × intensity) in 79 primary OVCA samples and 41 OVCA cell lines indicated the presence of c-Met expression in 92% (73/79) (Table I) and 83% (34/41) (Table II) of samples, respectively. c-Met expression in primary OVCA did not appear to be associated with stage, response to primary platinum therapy, or CA125 levels (Table I).

### Inhibition of HGF/c-Met signaling has in vitro anti-proliferative effects on OVCA cells and synergizes with combination carboplatin plus paclitaxel

**Figure 1:**
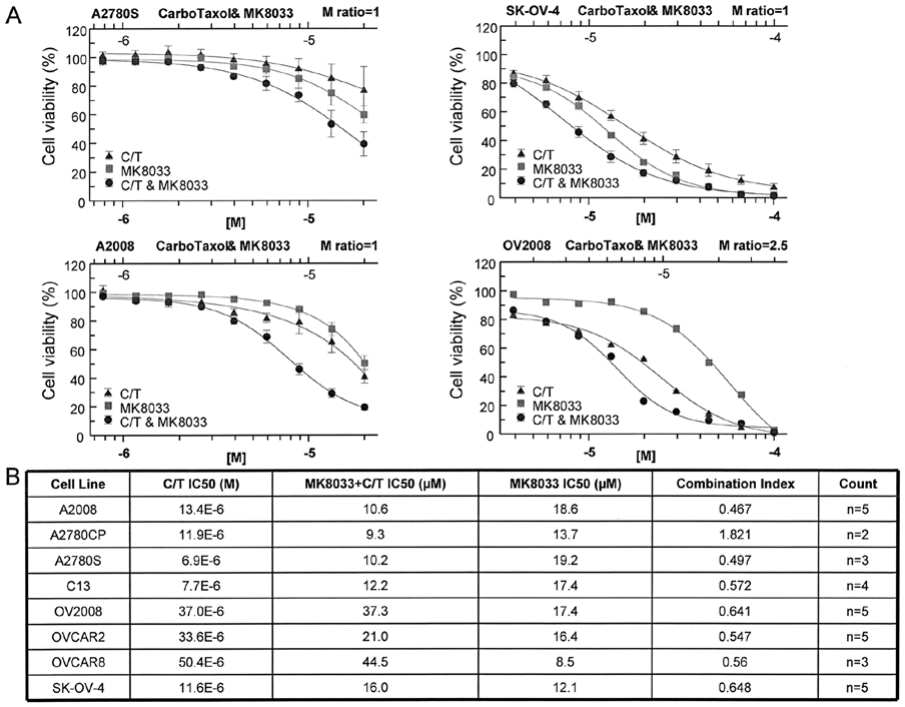
MK8033 acts synergistically with carboplatin plus paclitaxel (CarboTaxol or C/T) to inhibit OVCA cell line proliferation. (A) Line graphs show percent OVCA cell viability after 72-h incubation with MK8033, carboplatin plus paclitaxel (carboplatin-to-paclitaxel molar ratio of 20,000:1), and MK8033 plus carboplatin-paclitaxel at constant ratios of 1–2.5:1. Bottom and top horizontal axes indicate MK8033 and carboplatin (in carboplatin-paclitaxel mixture) concentrations, respectively. (B) IC_50_ values of carboplatin-paclitaxel, MK8033 plus carboplatin-paclitaxel, and MK8033 as well as combination index values of synergistic activity in OVCA cell lines. Count (n) refers to the number of replicate experiments.

To determine whether HGF signaling influenced OVCA cell line chemosensitivity, we evaluated the activity of MK8033, an inhibitor of HGF/c-Met signaling. MK8033 exhibited strong anti-proliferative effects against a panel of OVCA cell lines (Fig. 1). Furthermore, when combined in constant molar ratio of 1 with carboplatin plus paclitaxel (molar ratio of 20,000:1), MK8033 significantly lowered the carboplatin-paclitaxel IC_50_ in the majority of cell lines tested (Fig. 1). Combination index values calculated using the Chou-Talalay isobologram equation indicated synergistic activity between MK8033 and carboplatin-paclitaxel for almost all OVCA cell lines tested, as indicated by combination index values of <0.7 (Fig. 1).

### Molecular determinants of MK8033-carboplatin-paclitaxel sensitivity influence overall survival from OVCA

**Figure 2:**
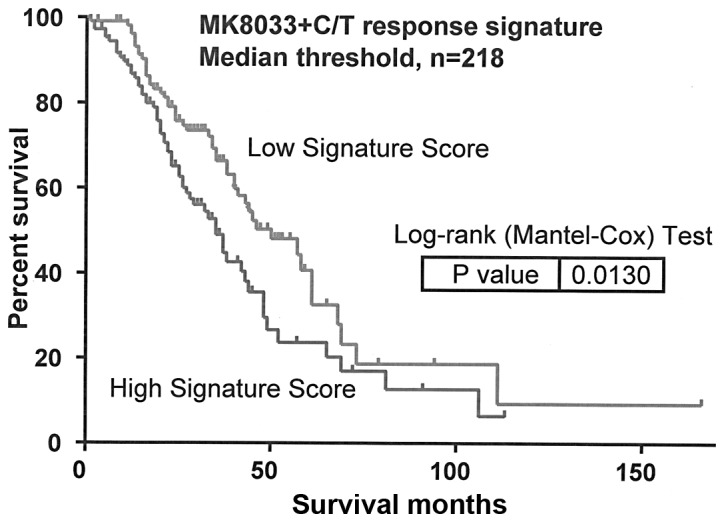
MK8033 plus carboplatin-paclitaxel (C/T) response signature may influence overall survival from OVCA. Kaplan-Meier curves depict the association between the expression of the 47-gene MK8033 plus carboplatin-paclitaxel response signature and overall survival from OVCA. Log-rank test P-values indicate significance. Analyses used the publicly available Australian Dataset (Affymetrix U133Plus GeneChips, n=220), with survival information available for 218 of the 220 samples.

Pearson's correlation test of IC_50_ values and Affymetrix HuRSTA genechip expression data identified 47 genes to be correlated with MK8033 plus carboplatin-paclitaxel sensitivity [false discovery rate (FDR) of <0.2] (Table III). PCA modeling of these genes indicated that the expression signature was associated with overall survival from OVCA (AUS dataset, n=218, P=0.013) (Fig. 2).

## Discussion

HGF is a paracrine growth factor originally found to be associated with liver regeneration (16–18). HGF signaling orchestrates a multitude of biological functions during embryogenesis, including cellular migration, invasion, proliferation, angiogenesis, wound healing, and tissue repair (19–29), and has been further associated with the development or progression of several different types of cancer, including OVCA (6,7,12,30–45). In OVCA, increased levels of HGF or overexpression of the HGF receptor c-Met has been associated with poor clinical outcome (5–7,16–18).

We found a near ubiquitous expression of c-Met in both primary OVCA and OVCA cell lines. To better understand the importance of HGF signaling in OVCA, we evaluated the activity of the novel inhibitor of HGF/c-Met signaling, MK8033, alone and in combination with carboplatin plus paclitaxel in a panel of OVCA cell lines (n=8). Although MK8033 showed anti-proliferative effects as a single agent, when used in a constant ratio to carboplatin-paclitaxel, the isobologram analysis indicated synergistic activity in the inhibition of OVCA cell proliferation. Pearson's correlation of cell line gene expression data and IC_50_ values identified the expression of 47 genes to be correlated with sensitivity to MK8033 plus carboplatin-paclitaxel. As shown by PCA modeling, genes correlated with MK8033 plus carboplatin-paclitaxel response were associated with overall survival from OVCA, suggesting that these genes may have a biological influence on OVCA response to therapy. Further analysis of the MK8033 plus carboplatin-paclitaxel response signature identified 6 of 47 genes to be significantly correlated with OVCA survival, including i) HFE (+ correlation, P=0.02); ii) UBQLN1 (+ correlation, P=0.04); iii) CLTCL1 (- correlation, P=0.04); iv) LPHN3 (- correlation, P=0.04); v) OSBPL6 (- correlation, P=0.03); and vi) SGK269 (- correlation, P=0.04).

The products of several of these genes have previously been implicated in the development and/or progression of cancer. For example, the product of the HFE gene, hemochromatosis protein, is involved in the regulation of iron absorption (46). Mutations in HFE and deregulation of iron absorption have been linked to liver cirrhosis and colon cancer risk (47,48). Ubiquilin 1, encoded by the UBQLN1 gene, has been shown to be associated with lung adenocarcinoma (49) and may affect OVCA response to metallodrugs (50). CLTCL1 encodes the heavy chain of clathrin, and the expression and/or activity of clathrin has been found to be associated with bladder cancer (51), TRAIL-resistant breast cancer (52), and uptake of some chemotherapeutic agents (53). Furthermore, the oxysterol binding protein (OSBP)-related protein 6, encoded by the OSBPL6 gene, belongs to a family of proteins recently identified as receptors for several natural products, including cephalostatin-1, ritterazine B, schweinfurthin A and OSW-1 (54).

In summary, we showed that targeted inhibition of HGF/c-Met signaling using the c-Met specific inhibitor, MK8033, worked synergistically with combinations of carboplatin plus paclitaxel to induce OVCA cell growth arrest. Furthermore, we demonstrated that genes associated with response to MK8033 plus carboplatin-paclitaxel may influence overall survival from OVCA, based on PCA modeling. The results of this study suggest that the inhibition of HGF/c-Met signaling may be a beneficial addition to the OVCA standard of care regimen of carboplatin plus paclitaxel therapy.

## Figures and Tables

**Table I:** c-Met expression in ovarian cancer samples and available clinical data.

Sample	Diagnosis	Score	CR vs. IR	Debulking	Age (years)	Stage	CA125
1	Focal adenocarcinoma	6	CR	O	57	IIIC	541
2	Papillary serous adenocarcinoma	1	CR	O	84	IIIC	7350
3	Adenocarcinoma	2	CR	O	53	IIIC	
4	Papillary serous adenocarcinoma	4	CR	O	61	IIIC	
5	Papillary serous adenocarcinoma	6	CR	O	57	IIIC	
6	Papillary serous adenocarcinoma	1	CR	O	55	IV	
7	Papillary serous adenocarcinoma	2	CR	O	49	IIIC	305
8	Papillary serous adenocarcinoma	1	CR	O	65	IIIC	
9	Papillary serous adenocarcinoma	4	CR	O	60	IIIC	925
10	Papillary serous adenocarcinoma	2	CR	O	51	IIIB	
11	Papillary serous adenocarcinoma	4	CR	O	56	IIIC	316
12	Papillary serous adenocarcinoma	2	CR	O	66	IIIC	224
13	Papillary serous adenocarcinoma	2	CR	S	56	IIIC	
14	Papillary serous adenocarcinoma	4	CR	O	73	IIIC	
15	Papillary serous adenocarcinoma	1	CR	O	62	IIIC	1717
16	Papillary serous adenocarcinoma	2	CR	O	78	IIIC	
17	Papillary serous adenocarcinoma	6	CR	O	43	IIIC	64
18	Papillary serous adenocarcinoma	1	CR	O	45	IIIC	
19	Papillary serous adenocarcinoma	1	CR	S	74	IIIC	1800
20	Papillary serous adenocarcinoma	2	CR	O	76	IIIC	
21	Papillary serous adenocarcinoma	2	IR	S	79	IV	
22	Papillary serous adenocarcinoma	2	IR	S	71	IV	1636
23	Papillary serous adenocarcinoma	2	CR	O	56	IIIC	
24	Papillary serous adenocarcinoma	1	CR	O	81	IIIC	
25	Papillary serous adenocarcinoma	2	CR	O	56	IIIC	260
26	papillary serous adenocarcinoma	3	CR	O	35	IV	47
27	Papillary serous adenocarcinoma	2	CR	O	53	IIIA	
28	Papillary serous adenocarcinoma	1	CR	S	77	IV	>600
29	Papillary serous adenocarcinoma	2	CR	O	65	IIIC	1118
30	Papillary serous adenocarcinoma	1	CR	S	47	IIIC	712
31	Papillary serous adenocarcinoma	2	CR	S	76	IIIC	1848
32	Papillary serous adenocarcinoma	2	CR	O	70	IIIC	
33	Papillary serous adenocarcinoma	3	CR	S	57	IIIC	266
34	Adenocarcinoma metastatic	2	CR	O	57	IIIC	175
35	Papillary serous adenocarcinoma	1	CR	O	65	IV	404
36	Papillary serous adenocarcinoma	2	CR	O	76	IV	
37	Papillary serous adenocarcinoma	1	CR	O	66	IIIC	
38	Papillary serous adenocarcinoma	1	CR	O	68	IIIC	
39	Papillary serous adenocarcinoma	3	IR	O	73	IIIC	
40	Papillary serous adenocarcinoma	2	CR	O	63	IV	
41	Papillary serous adenocarcinoma	2	IR	S	63	IIIC	
42	Papillary serous adenocarcinoma	2	IR	O	47	IIIC	
43	Papillary serous adenocarcinoma	2	CR	O	42	IIIC	110
44	Papillary serous adenocarcinoma	1	CR	O	74	IIIC	4557
45	Papillary serous adenocarcinoma	2	CR	S	64	IIIC	
46	Papillary serous adenocarcinoma	2	IR	S	64	IIIC	456
47	Papillary serous adenocarcinoma	1	IR	O	71	IIIC	
48	Papillary serous adenocarcinoma	6	IR	O	69	IIIC	
49	Papillary serous adenocarcinoma	3	CR	O	49	IIIC	
50	Papillary serous adenocarcinoma	2	CR	O	62	IV	
51	Papillary serous adenocarcinoma	2	IR				
52	Focal adenocarcinoma	3	CR	S	88	IIIC	
53	Serous adenocarcinoma	1	IR	O	74	IIIC	101
54	Papillary serous adenocarcinoma	2	IR	O	71	IIIC	
55	Papillary serous adenocarcinoma	4	IR	O	69	IIIC	1606
56	Papillary serous adenocarcinoma	1	IR	O	52	IIIC	
57	Papillary serous adenocarcinoma	2	CR	O	67	IIIC	
58	Papillary serous adenocarcinoma	1	CR	O	66	IIIC	824
59	Papillary Serous adenocarcinoma	2	IR	O	52	IIIC	
60	Papillary serous adenocarcinoma	1	CR	S	73	IIIC	2354
61	Papillary serous adenocarcinoma	0	CR	O	75	IIIC	
62	Papillary serous adenocarcinoma	2	IR	O	65	IIIC	
63	Focal cellular atypia	n/a	CR	O	74	IIIC	
64	Papillary serous adenocarcinoma	2	IR	O	79	IIIC	417
65	Papillary serous adenocarcinoma	1	CR	O	73	IIIC	180
66	Papillary serous adenocarcinoma	2	IR	O	53	IV	96
67	Papillary serous adenocarcinoma	1	CR	O	60	IIIC	
68	Papillary serous adenocarcinoma	2	CR				
69	Papillary serous adenocarcinoma	2	IR	S	53	IIIC	
70	Papillary serous adenocarcinoma	1	IR	O	41	IIIC	2800
71	Papillary serous adenocarcinoma	1	CR	O	80	IIIC	
72	Papillary serous adenocarcinoma	2	CR	O	42	IIIA	
73	Papillary serous adenocarcinoma	2	IR	S	66	IIIC	90
74	Papillary serous adenocarcinoma	1	CR	S	60	IIIC	750
75	Papillary serous adenocarcinoma	4	CR	O	77	IIIC	9814
76	Papillary serous adenocarcinoma	0	CR	O	72	III	
77	Papillary serous adenocarcinoma	1	IR	O	66	IIIC	
78	Papillary serous adenocarcinoma	0	CR	O	54	III	
79	Papillary serous adenocarcinoma	3	CR	O	38	IIIC	

The c-Met expression score was determined by intensity × cellularity, where intensity was graded as 1, weak; 2, moderate; or 3, strong, and cellularity was graded as 1 when ≤33%, 2 when 34–65%, or 3 when ≥66%. CR, complete response; IR, incomplete response to primary therapy.

**Table II:** c-Met expression in ovarian cancer cell lines.

Cell line	Cellularity	Intensity	Score
A2008	2	2	4
A2780CP	0	0	0
A2780S	1	1	1
BGI	0	0	0
C13	3	1	3
CAOV3	3	1	3
CHI	0	0	0
CHI cisR	1	1	1
CAOV2	3	2	6
Dov 13	3	2	6
HeyA8	3	1	3
IGR-OV1	3	2	6
IMCC3	2	1	2
IMCC5	1	1	1
M41	1	1	1
M41CSR	2	1	2
MCAS	3	1	3
OV2008	1	1	1
OV90	1	1	1
Ovary1847	1	1	1
OVCA 429	2	1	2
OVCA 432	Acellular	n/a	n/a
OVCA 433	3	1	3
OVCA420	1	2	2
OVCAR10	0	0	0
OVCAR2	3	2	6
OVCAR3	2	1	2
OVCAR4	3	2	6
OVCAR5	3	2	6
OVCAR8	3	1	3
PEO1	3	2	6
PEO4	2	2	4
SKOV8	2	1	2
SKOV3	3	2	6
SKOV4	0	0	0
SKOV6	2	1	2
T8	3	2	6
Tov-112D	2	1	2
Tov-21-G	1	1	1
Tyknu	0	0	0
Tyknu CisR	0	0	0

The c-Met expression score was determined by intensity × cellularity, where intensity was graded as 1, weak; 2, moderate; or 3, strong, and cellularity was graded as 1 when ≤33%, 2 when 34–65% or 3 when ≥66%.

**Table III:** Genes associated with ovarian cancer cell sensitivity to MK8033 plus carboplatin-paclitaxel.

Signature gene symbols			
SETD6	UBQLN1	HDAC3	LOC653441
RNF207	NDOR1	CLTCL1	PHC1
ABCC8	IGHM	PPIL4	MATR3
DNASE1L1	FBXO4	REST	CLYBL
ACD	PRKAA1	LPHN3	MLLT4
NUP155	RIPK4	DKFZp667	EFCBP2
FAM91A2	TASP1	ACTN2	KIAA0528
FAM91A1	ADRBK2	OSBPL6	PPM1J
MGC5566	FAM46C	STAR	TMEFF1
MRPL30	ITIH4	FUNDC2	SGK269
MAGIX	C1orf164	RAB15	LOC389634
HFE	IL17RD	EDA	
